# Diagnostic trap: a case report of intimal sarcoma occurring in the left atrium

**DOI:** 10.3389/fcvm.2025.1509505

**Published:** 2025-02-13

**Authors:** Hua Ye, Yuchen Jing, Shuai Luo, Jinjing Wang

**Affiliations:** Department of Pathology, Affiliated Hospital of Zunyi Medical University, Zunyi, Guizhou, China

**Keywords:** diagnosis, immunotherapy, intimal sarcoma, left atrium, MDM2, vascular

## Abstract

**Background:**

Intimal Sarcoma (IS) is an exceptionally rare and highly aggressive mesenchymal tumor with an uncertain origin. Its clinical and pathological characteristics are challenging to differentiate from other tumors based merely on histological and cytological morphology. Additionally, the immunohistochemical phenotype lacks specificity. Genomically, IS is distinguished by the amplification of the Mouse Double Minute 2 homolog (MDM2) gene. Presently, there are significant obstacles in clinical diagnosis and differential diagnosis of this condition.

**Case demonstration:**

A 49-year-old male patient was hospitalized due to cough and dyspnea. An echocardiogram indicated a myxoma, leading to the performance of a partial cardiac tumor resection. Post-surgical pathological analysis revealed numerous spindle-shaped tumor cells organized in bundles. The cells displayed significant atypia, areas of necrosis, myxoid degeneration, and pathological mitotic figures. Immunophenotyping indicated positivity for Vimentin, Smooth Muscle Actin, and MDM2, focal positivity for ETS-Related Gene, and a Ki-67 index of 40%, with other markers being negative. Fluorescence *in situ* Hybridization genetic testing confirmed MDM2 gene amplification. The diagnosis was established as IS of the left atrium, World Health Organization grade 2. Post-surgery, six cycles of chemotherapy were administered. An 11-month follow-up period revealed tumor recurrence and progression, with multiple lesions but no distant metastases.

**Conclusions:**

A rare case of cardiovascular IS located in the left atrium has been documented. Diagnosing this condition poses significant challenges based solely on histological, cytomorphological, and immunophenotypic characteristics, as differentiation from angiosarcoma, malignant mesothelioma, synovial sarcoma, and myxofibrosarcoma is difficult. Consequently, diagnosing IS necessitates a comprehensive approach that integrates clinical presentation, echocardiography, and pathological examinations, encompassing morphology, immunohistochemistry, and genomic analysis. Surgical resection remains the primary treatment option. However, the rate of postoperative recurrence is high, and the prognosis remains poor. Adjuvant chemotherapy and radiotherapy are suggested. In advanced cases, comprehensive immunotherapy methods may be employed to enhance patient survival rates and quality of life.

## Background

Cardiac tumors are exceedingly rare, with malignant variants having an even lower incidence, constituting approximately 10% of all cardiac tumors (1, 2). Among primary malignant cardiac tumors, 95% are sarcomas (3). The World Health Organization (WHO) (2015) histopathological classification of cardiac tumors categorizes the histological subtypes of cardiac sarcomas into angiosarcoma, fibrosarcoma, synovial sarcoma, leiomyosarcoma, rhabdomyosarcoma, malignant fibrous histiocytoma, undifferentiated sarcoma, and others. Intimal sarcoma (IS) is classified as a malignant tumor with uncertain differentiation, with its pathogenesis remaining unclear and possibly originating from multipotent mesenchymal cells (4).

Additionally, IS is an exceptionally rare and highly malignant mesenchymal tumor of unknown origin, typically occurring in the intima of large vessels within the systemic or pulmonary circulation (5). It is currently hypothesized to originate from subendothelial intimal mesenchymal cells (6), proliferating into the lumen and locally invading the vessel wall. Furthermore, it can also form tumor emboli that obstruct blood vessels or metastasize distally along small pulmonary arteries (7). Most ISs are poorly differentiated, with immunohistochemical and electron microscopic studies suggesting fibroblastic or myofibroblastic differentiation. A rare instance of cardiovascular IS occurring in the left atrium is documented here, characterized by an acute onset and non-specific clinical manifestations. Due to the rarity of this condition, most information is derived from individual pathological reports.

## Case demonstration

**Figure 1:**
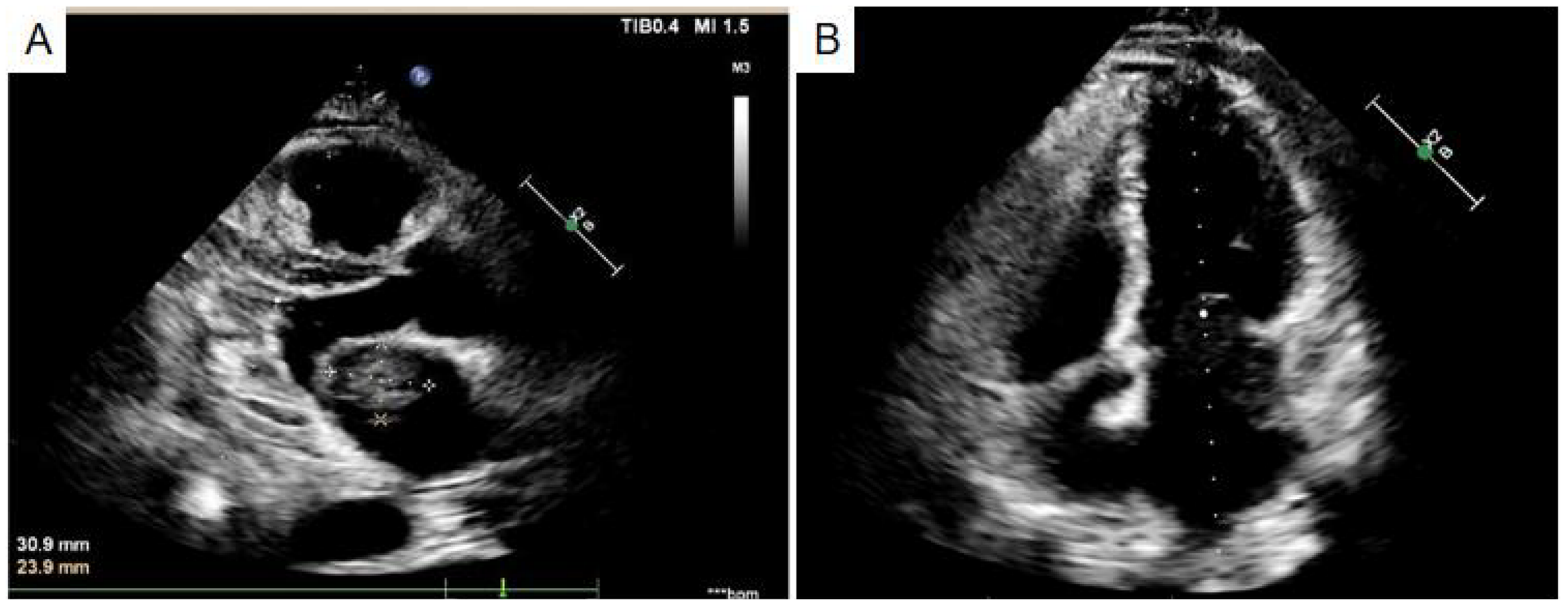
Echocardiography indicated a left atrial mass **(A)**, suggestive of myxoma, obstructive mitral stenosis **(B)**.

A 49-year-old male patient was hospitalized presenting a 20-day history of cough and shortness of breath, which had exacerbated over the past week. The initial diagnosis indicated small pleural effusion and pneumonia. Oral medication did not lead to any improvement, and there was no significant change in weight. Auscultation revealed scattered moist rales in both lungs without wheezing. Heart sounds were normal, with no enhancement or weakening. No additional heart sounds or murmurs were detected in the valve areas, and there was no pericardial friction rub. Palpation revealed mild tenderness below the xiphoid process without rebound tenderness or muscle tension. Symptomatic treatment, including cough suppression and anti-infection therapy, was administered. Ultrasound examination and thoracocentesis and paracentesis and abdominal effusion indicated bilateral pleural effusion (left side encapsulated). A chest thin-layer computed tomography (CT) scan with 3D reconstruction revealed bilateral pleural effusion, small pericardial effusion, and multiple enlarged mediastinal lymph nodes. Elevated levels of brain natriuretic peptide (BNP) were observed, with N-terminal pro-B-type natriuretic peptide at 1,016.2 pg/ml. Echocardiography (Figure 1) indicated a left atrial mass, suggestive of myxoma, obstructive mitral stenosis, mild tricuspid regurgitation, pulmonary hypertension, and small pericardial effusion. Global heart failure was assessed as class II-III (according to the New York Heart Association). Coronary CT angiography showed mild stenosis in the middle segment of the left anterior descending artery, close to the interventricular groove.

The patient's condition was critical, necessitating continuous oxygen therapy and cardiac monitoring. A median sternotomy was performed to partially resect the cardiac tumor with the assistance of cardiopulmonary bypass during open heart surgery. Intraoperative exploration revealed a solid mass, approximately 40 mm × 30 mm, in the left atrium, attached to the orifice of the left atrial appendage. Multiple nodules of varying sizes, with the largest measuring about 10 mm × 5 mm, were observed on the left atrial wall and the posterior leaflet of the mitral valve. The solid mass exhibited a fish-flesh appearance upon cross-section. Based on intraoperative findings, malignancy was suspected. An intraoperative frozen section biopsy suggested a malignant tumor of mesenchymal origin. Complete tumor resection was not feasible during surgery, and the metastatic lesions on the left atrial wall and mitral valve could not be removed.

### Pathological examination

Gross examination: A grayish-white mass of tissue, measuring 40 mm × 40 mm × 10 mm, was noted. The cut surface was grayish-white, solid, and had medium consistency.

**Figure 2:**
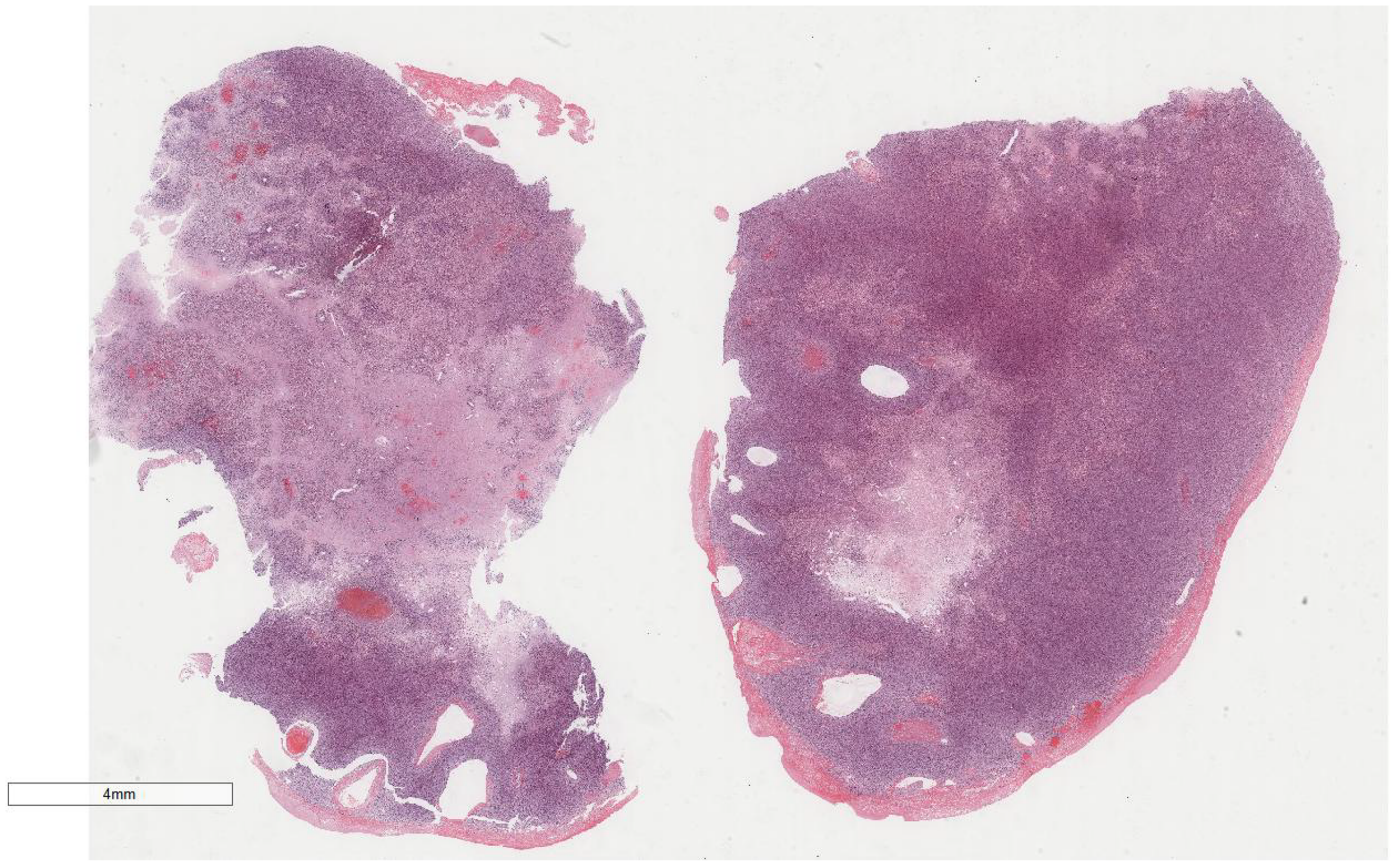
At low magnification, the tumor was elliptic and the boundary was clear. H&E × 5.

**Figure 3:**
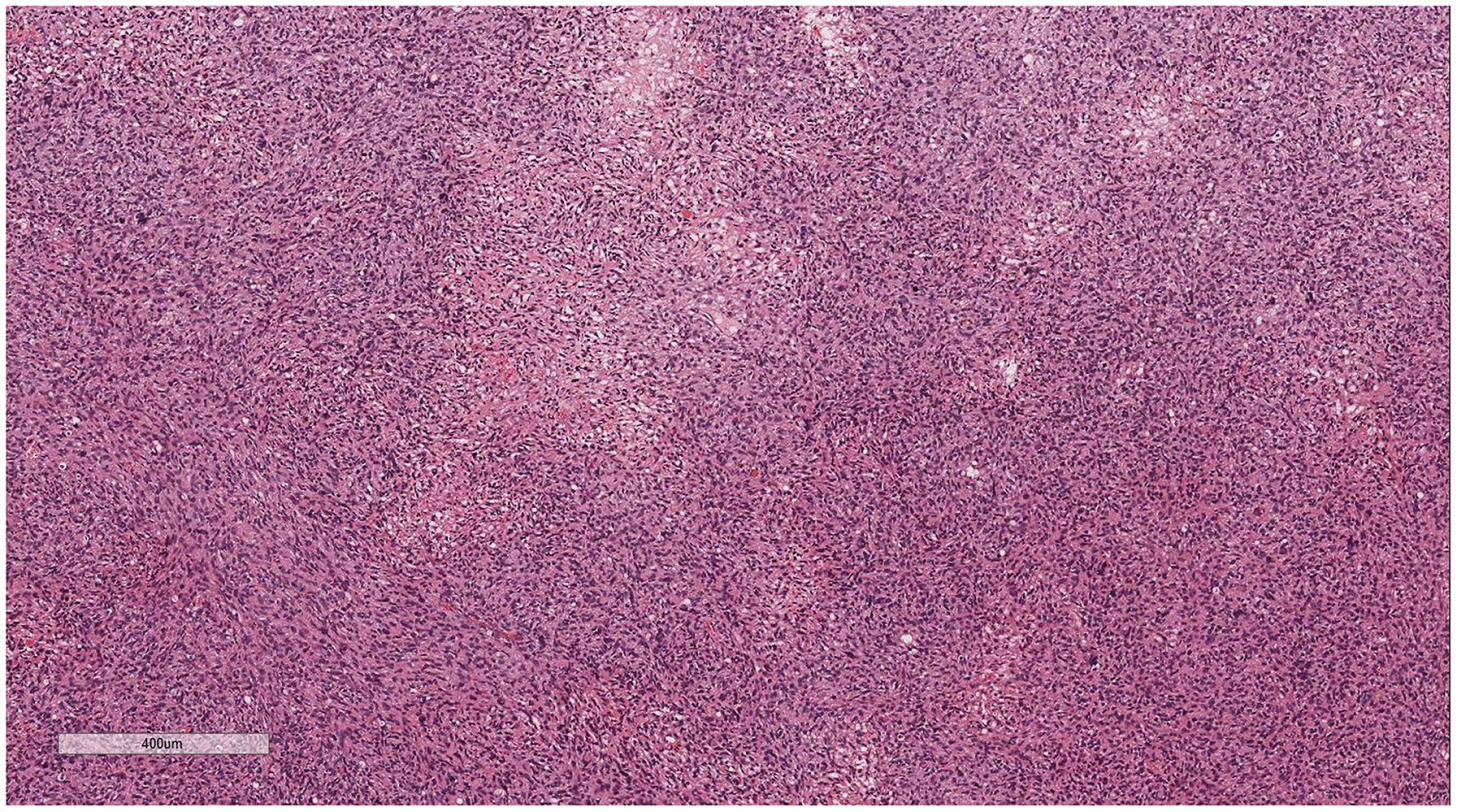
Numerous spindle-shaped tumor cells were seen, arranged in fascicular or interlacing patterns. H&E × 50.

**Figure 4:**
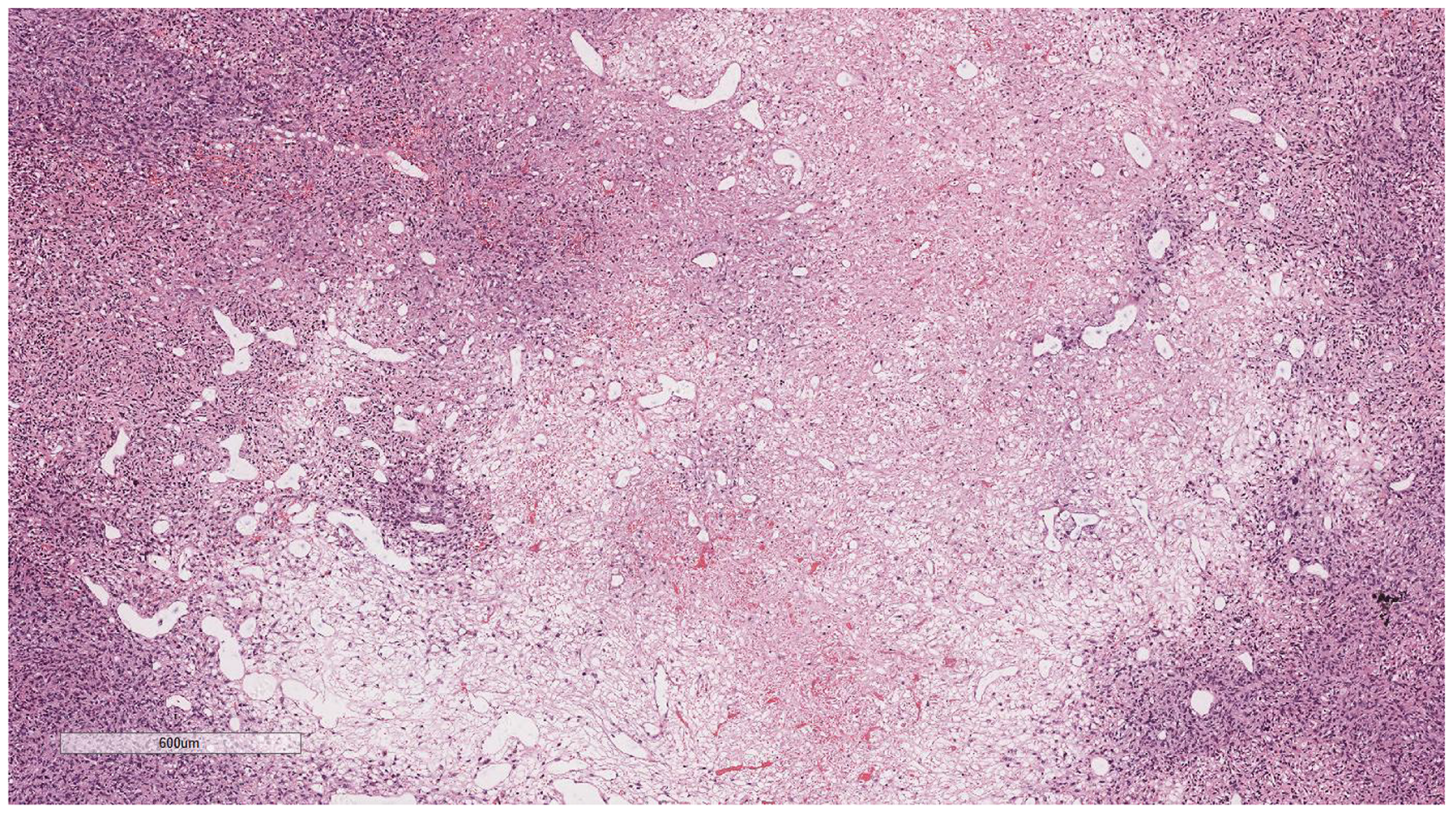
Focal necrosis and myxoid degeneration were present. H&E × 40.

**Figure 5:**
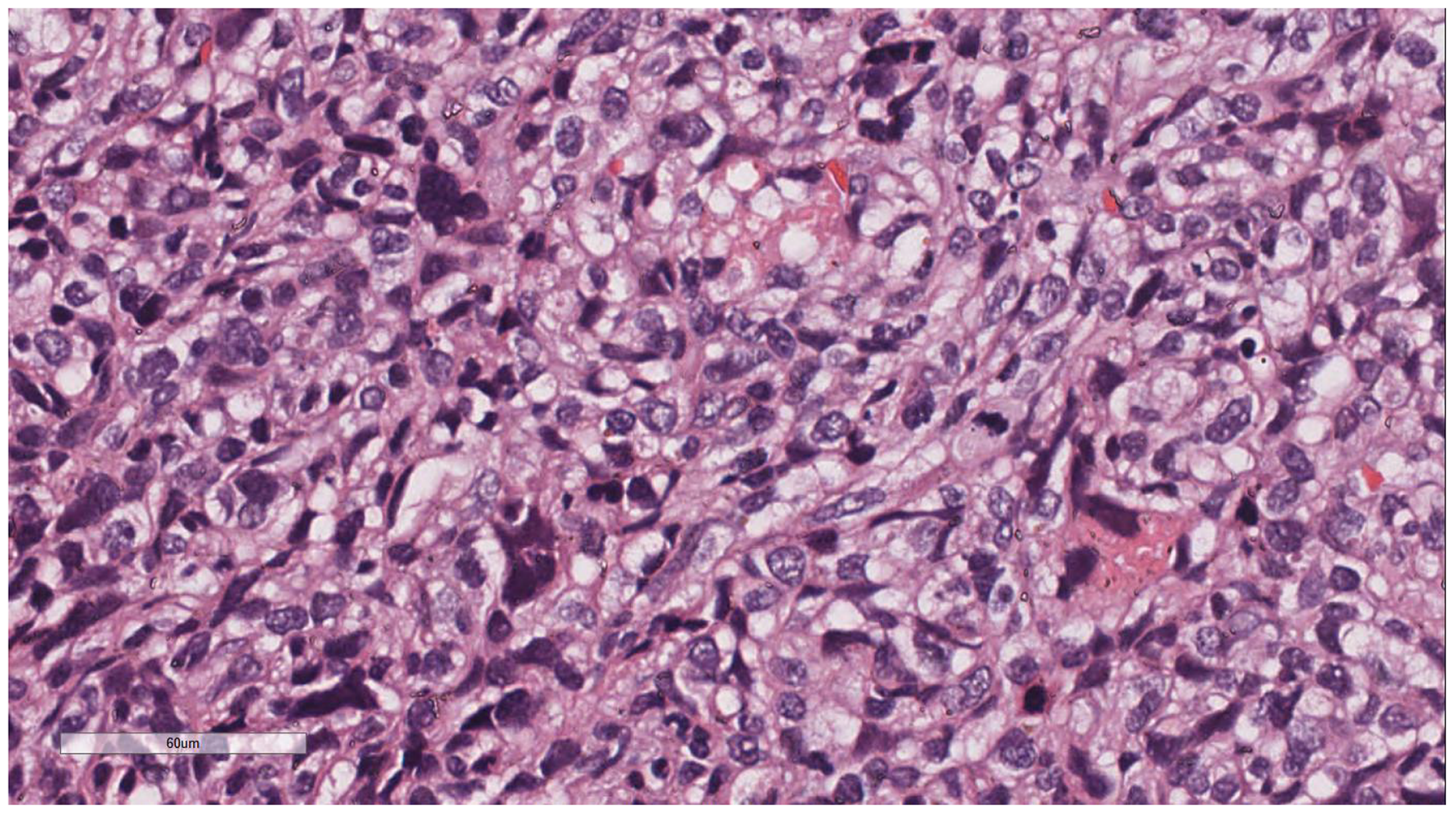
Multinucleated tumor giant cells were identified in some regions. Mitotic figures were frequently observed, with 4–10 per 10 high-power fields (HPF), including atypical mitoses. H&E × 400.

Microscopic examination (Figure 2): Microscopically, numerous spindle-shaped tumor cells were seen, arranged in fascicular or interlacing patterns (Figure 3). These cells showed significant atypia, with prominent nucleoli and abundant eosinophilic cytoplasm. Focal necrosis and myxoid degeneration were present (Figure 4). The stroma was relatively abundant and exhibited increased vascularity. Multinucleated tumor giant cells were identified in some regions. Mitotic figures were frequently observed, with 4–10 per 10 high-power fields (HPF), including atypical mitoses (Figure 5).

**Figure 6:**
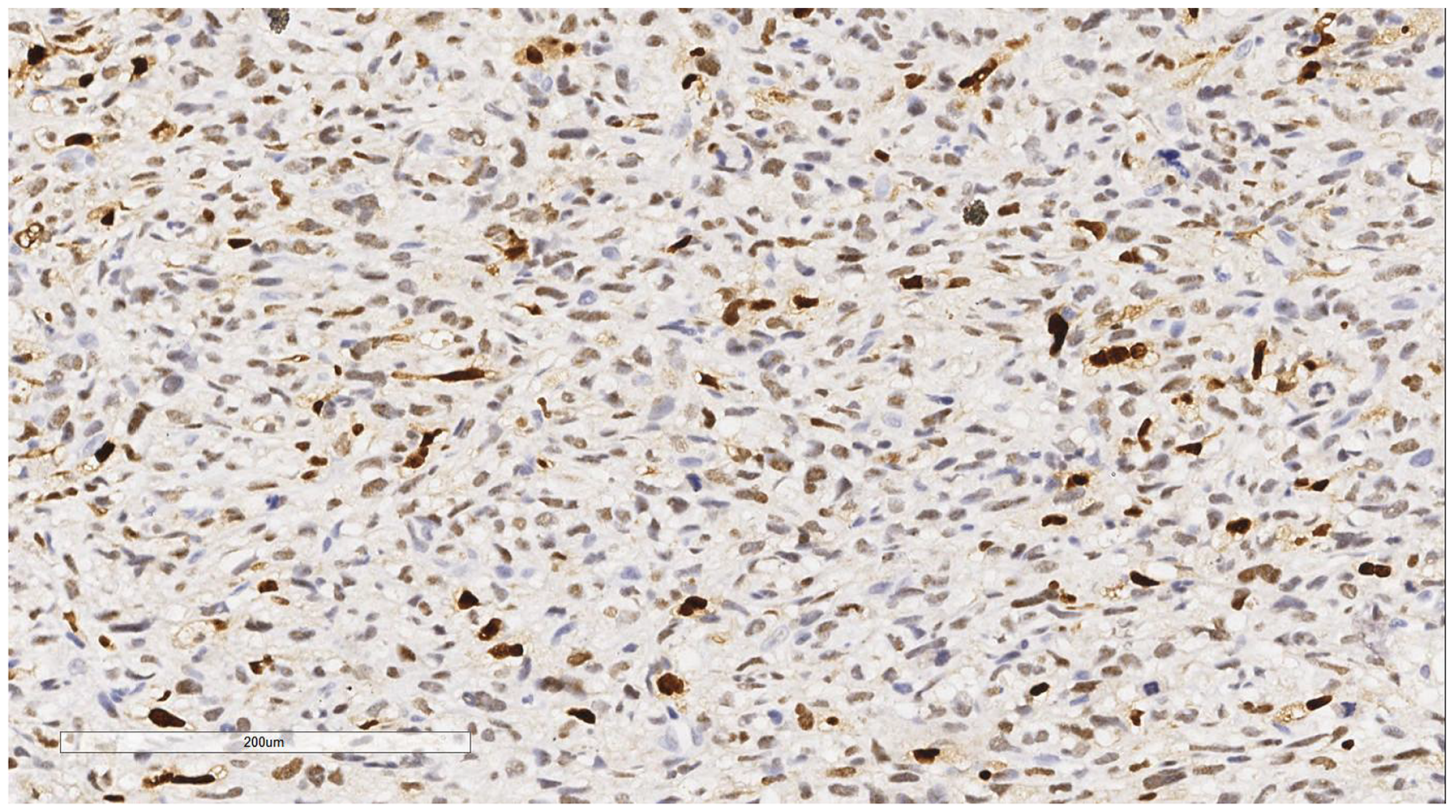
Immunohistochemistry reveals a tumor cells MDM2 (+). EnVision, ×200.

**Figure 7:**
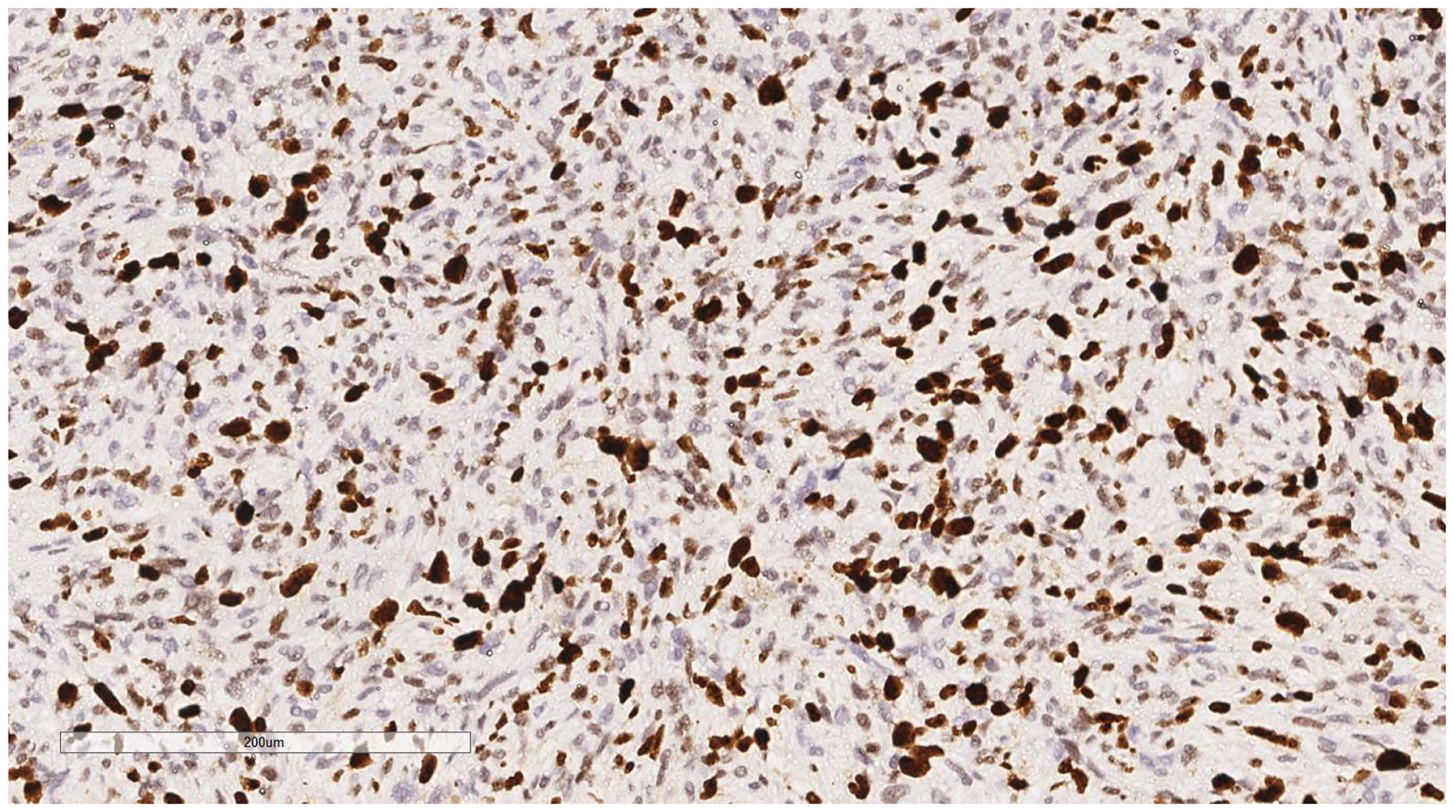
Immunohistochemistry reveals a tumor cells Ki-67 proliferation index (40%, +). EnVision, ×200.

**Figure 8:**
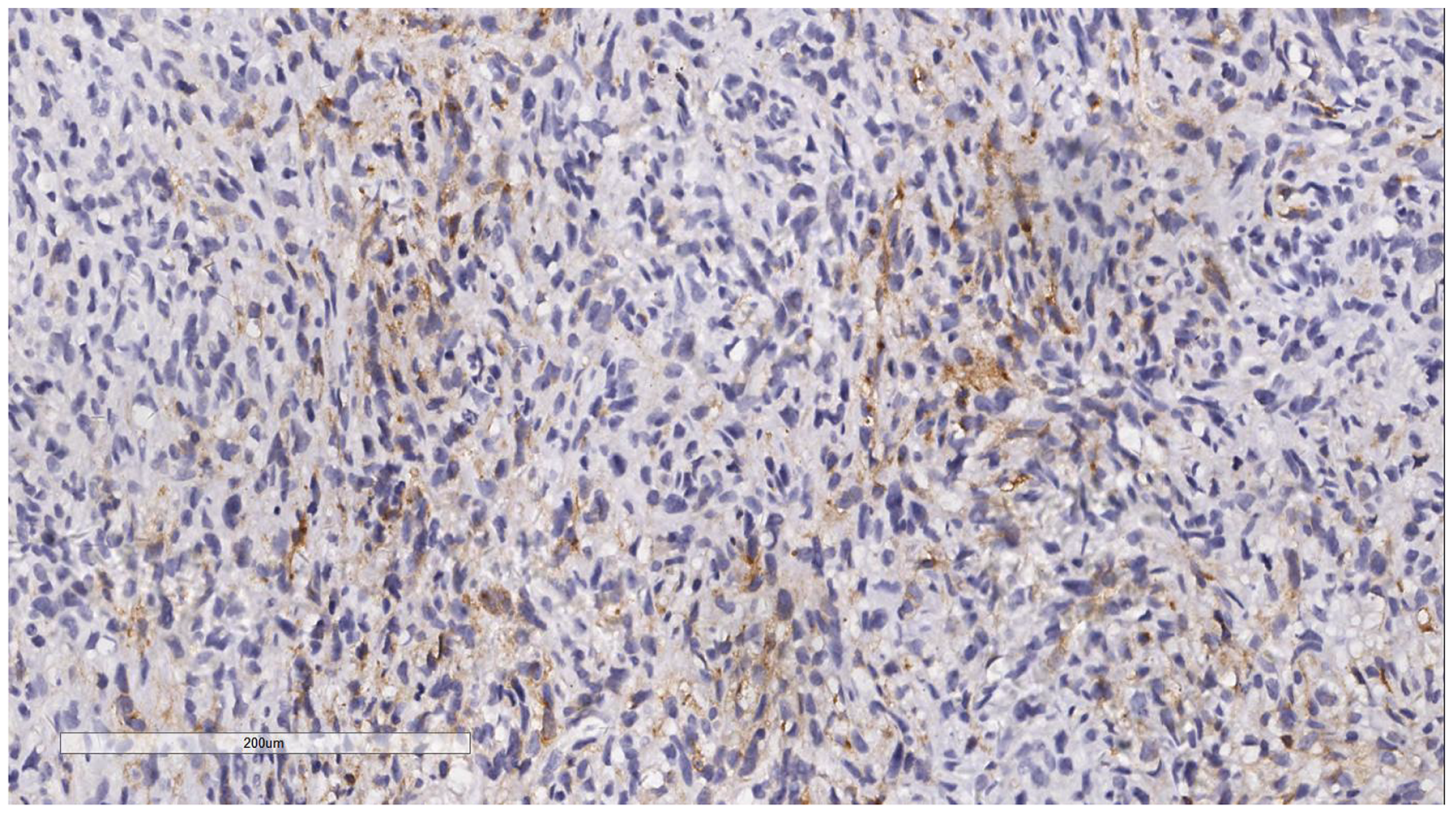
Immunohistochemistry reveals a tumor cells PD-L1 (22C3): 1 point. EnVision, ×200.

Immunophenotype: Vimentin (+), Smooth Muscle Actin (SMA) (+), Mouse Double Minute 2 homolog (MDM2) (+) (Figure 6), ETS-related gene (ERG) (focally +), Epithelial Membrane Antigen (EMA) (—), Desmin (—), Cluster of Differentiation 31 (CD31) (—), Cluster of Differentiation 34 (CD34) (—), S100 protein (S-100) (—), Ki-67 (Figure 7) proliferation index (40%, +), PD-L1 (22C3):1 point (Figure 8).

**Figure 9:**
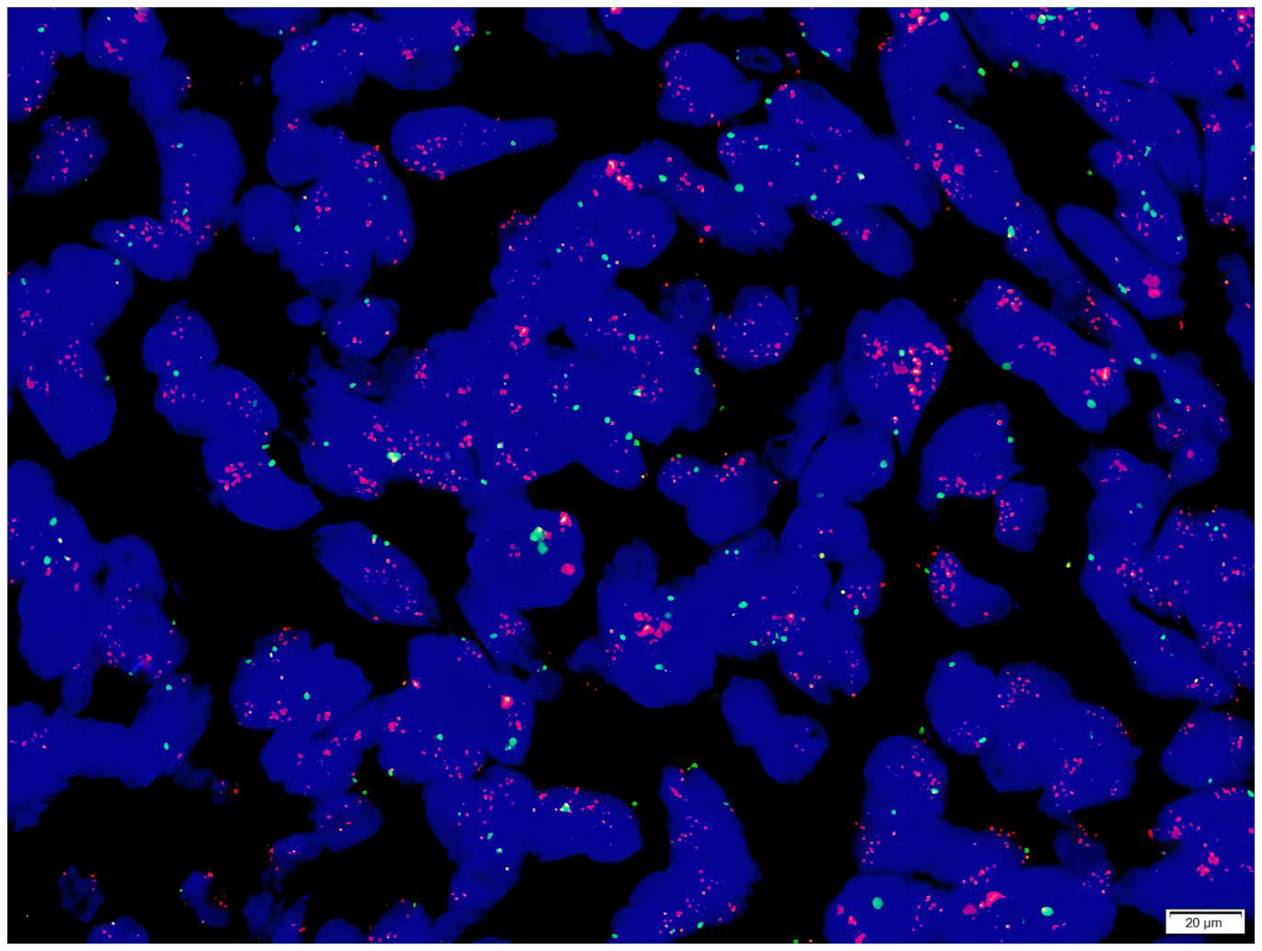
The average ratio of MDM2/CEP12 signals was calculated as 5.02 (>2). The MDM2 amplification status, determined by FISH (fluorescence *in situ* hybridization), was confirmed to be positive.

Gene detection (Figure 9): A dual-color probe of MDM2/Chromosome Enumeration Probe 12 (CEP12) was utilized, where orange-red signals (R) denoted the MDM2 gene and green signals (G) denoted CEP12. Fifty cells were counted. The MDM2 signals totaled 617, averaging 12.34 per cell, while the CEP12 signals totaled 123, averaging 2.46 per cell. The average ratio of MDM2/CEP12 signals was calculated as 5.02 (>2). The MDM2 amplification status, determined by FISH (fluorescence *in situ* hybridization), was confirmed to be positive.

Pathological diagnosis: IS of the left atrium, WHO grade 2.

Postoperatively, intensive care and specialized nursing were provided. Dynamic blood gas analysis was conducted, and measures to prevent incision infection were implemented. Symptomatic supportive treatments were administered, including hemostasis, acid suppression, expectoration, and myocardial nutrition. Vasoactive drugs were utilized to maintain circulatory stability. Five days post-surgery, a chest CT scan revealed postoperative changes in the sternum, small fluid accumulation in the anterior mediastinum and pericardium, pneumonia in both lungs with partial atelectasis in the lower lobes, small bilateral pleural effusions, and enlarged mediastinal lymph nodes. Echocardiography indicated post-partial cardiac tumor resection status with an irregular echo measuring approximately 15 mm × 8 mm near the left atrial appendage, without significant movement. Mild regurgitation of the mitral, tricuspid, and pulmonary valves was observed. Left ventricular systolic function: left ventricular ejection fraction was 60% and fractional shortening was 32%. The electrocardiogram indicated atrial premature beats.

The patient underwent six cycles of chemotherapy within six months following surgery. Each month, a course of chemotherapy was administered, comprising “doxorubicin hydrochloride liposome 55.0 mg + ifosfamide 3.25 g + mesna 0.6 g”. Based on medical advice, the patient was admitted to the hospital for treatment of a “cardiac tumor” three months after the final chemotherapy session. The patient reported experiencing chest tightness and shortness of breath at rest, as well as a reduced quality of life. Cardiac ultrasound indicated a solid space-occupying lesion in the left atrium, multiple irregular solid echo masses on the left atrial wall, with the largest measuring approximately 44 mm × 25 mm, left atrial enlargement, poor mobility of the posterior mitral valve leaflet with slightly increased forward flow velocity at the valve orifice, and mild mitral and tricuspid regurgitation. Chest CT revealed proliferative foci, calcified foci, and multiple fibrotic foci in the upper and lower lobes of both lungs, with increased small lymph nodes in the mediastinum. The oncology department initially planned to administer second-line chemotherapy or immunotherapy according to guidelines. However, the patient reported a poor physical condition, making it difficult to tolerate further chemotherapy. Additionally, due to financial constraints, the patient was unable to afford immunotherapy costs and refused further anti-tumor treatment.

## Discussion

Primary malignant cardiac tumors are rare, typically occurring in any part of the heart, though they are more frequently found in the right cardiac system, particularly in the right atrium (8). The case presented in this study involves an IS in the left atrium, an exceedingly rare location. Patients with this condition generally report symptoms such as cough, chest tightness, and shortness of breath, which are often non-specific. A minority may exhibit signs of heart failure, including lower limb edema and dyspnea, complicating the differentiation from other cardiopulmonary diseases. By the time a definitive diagnosis is achieved, the disease has often advanced to the middle or late stages.

Cardiac color Doppler echocardiography can illustrate the movement of the tumor and its impact on hemodynamics, offering a valuable tool for early detection and definitive diagnosis of cardiac tumors (9). Cardiac computed tomography (CCT) can visually show the initial location, extent of the lesion and its adjacent relationship with the surrounding tissue, and observe the perfusion and density of the cardiac space-occupying lesion, which is helpful to evaluate the course of the adjacent coronary artery and pulmonary large vessels, and provide more comprehensive and accurate imaging information for surgery. Cardiac Magnetic Resonance Imaging (CMRI) can noninvasively evaluate the function of the left ventricle and each myocardial segment, display the myocardial perfusion and the location and area of myocardial infarction, and then judge the myocardial activity, which is of great significance in cardiac occupying lesions (10). In imaging, primary cardiac angiosarcoma shows homogeneous or heterogeneous density in plain CT images, and heterogeneous centritic enhancement in enhanced CT images, which can be initially differentiated from benign tumors such as myxoma (11).

In this case, a preoperative cardiac ultrasound revealed a mass in the left atrium, initially suspected to be a myxoma. Nevertheless, the pathological results from surgery remain the gold standard for definitive diagnosis. During partial resection of the cardiac tumor, a solid fish-flesh-like mass measuring approximately 40 mm × 30 mm was observed in the left atrium. Intraoperative frozen section pathology suggested a malignant tumor of mesenchymal origin. Under HE microscopy, the tumor cells appeared spindle-shaped, arranged in an interlacing or fascicular pattern, with significant atypia, distinct nucleoli, and abundant eosinophilic cytoplasm. Areas of patchy necrosis and myxoid degeneration were noted, along with the proliferation of interstitial blood vessels. Some regions showed multinucleated giant tumor cells, with frequent mitotic figures (4–10 per 10 HPF), including atypical mitoses. Immunohistochemical staining showed positivity for Vimentin, SMA, and MDM2, scattered positivity for ERG, negativity for EMA, Desmin, CD31, CD34, and S-100, and Ki-67 positivity in 40% of cells.

The following differential diagnoses should be considered (12):

(1) Angiosarcoma: Microscopically, spindle-shaped or ovoid tumor cells form irregular vascular lumina that interconnect and communicate. These spaces are typically lined by atypical endothelial cells, with occasional multinucleated forms observed. Immunohistochemical markers CD31, CD34, ERG, D2-40, and Factor VIII are positive.
(2) Malignant mesothelioma: Microscopic examination reveals epithelioid tumor cells in papillary or nodular patterns. Occasionally, the tumor displays biphasic characteristics, with spindle cells and mesothelial-like cells coexisting. These cells have abundant eosinophilic cytoplasm, large nuclei with a vesicular or hyperchromatic appearance, and no mitotic figures. Immunohistochemical markers Calretinin and CK5/6 are positive, while adenocarcinoma markers are negative.
(3) Synovial sarcoma: Microscopically, spindle-shaped tumor cells exhibit a biphasic pattern with alternating dense and edematous areas. The cells are small and compact, with occasional lymphocytic infiltration. Epithelioid cells are arranged in clusters or nests, with gland-like structures occasionally observed.
(4) Myxofibrosarcoma: Microscopically, sarcomas tend to have a spindle or short spindle cell pattern, which be asteroidal in mucous area. The nuclei presents rotundity and hyperchromatic,and mitotic figure is rare. Immunohistochemical markers MUC4, DOG1 are positive, CD99, BCL-2 are expressed occasionally, while Desmin, S-100, EMA, CD34 are negative. However, Intimal sarcoma is poorly differentiated malignant tumor, which is difficult to distinguish from other malignancies only in terms of tissue and cell morphology, and the immunohistochemical phenotype lacks specificity, vimentin 、SMA are positive, others are mostly negative (13). Therefore, in order to further clarify the diagnosis, we sought the help of genomics.

A review of domestic and international literature reveals that genomic studies on cardiac IS are scarce. Regarding molecular alterations, it has been clearly established that IS is characterized by MDM2 amplification (14). MDM2, a proto-oncogene located in the 12q12-15 region near the CDK4, GLI-1, DDIT3, and HMGA2 genes, functions as a negative regulator of TP53 (15). It encodes an oncoprotein acting as an E3 ubiquitin ligase, targeting the p53 protein for proteasomal degradation, thereby circumventing the regulatory effects of the tumor suppressor gene p53 on cell growth (16). Furthermore, reports have indicated that besides MDM2 gene amplification, IS may also exhibit amplifications of CDK4, PDGFRA, GLI-1, HMGA2, DDIT3, and KIT6 (12, 17). Some studies have identified deletions of TP53, RB1, PTEN, CDKN2A, and CDKN2B in IS (18), as well as loss of p16 expression, which may contribute to disease progression (19). In the present case, molecular testing revealed MDM2 gene amplification, providing a new therapeutic target for the patient. However, the possibility of additional gene therapy targets cannot be excluded.

Currently, no definitive effective treatment regimen exists for this disease, either domestically or internationally. Early diagnosis and surgery are recommended. For cases without distant metastasis, complete surgical resection is the primary choice. However, achieving complete surgical removal is challenging, and the postoperative recurrence rate is high, resulting in poor prognosis (20). Heart transplantation is considered for tumors that have not invaded the myocardial tissue (21). If treatment outcomes are unsatisfactory, the patient's survival period is conservatively estimated at only 6–18 months (22). In this case, the tumor could not be entirely removed during surgery, and metastases in the left atrial wall and mitral valve could not be excised. During an 11-month follow-up, the patient reported poor results after six rounds of chemotherapy, experiencing symptoms such as chest tightness and shortness of breath at rest, with a diminished quality of life. Cardiac color Doppler ultrasound showed enlarged and multiple tumors. Given the patient's condition, the oncology department suggested immunotherapy as a treatment approach.

PD-1 antibodies are currently the most widely used immune checkpoint therapy drugs in clinical practice (23). However, soft tissue sarcomas are immunologically characterized as “cold tumors,” and immune checkpoint inhibitors (ICIs) have not shown particularly promising efficacy for soft tissue sarcomas. Current clinical studies on immune checkpoint drugs (such as SARC028 and Alliance A091401) indicate that these drugs are only effective for certain soft tissue sarcomas (24, 25). In this case, the patient's PD-L1 (22C3) score combined with a positive score of 1. PD-1 monoclonal antibody treatment could be considered, but immune checkpoint therapy often takes effect slowly, while sarcomas progress rapidly. Therefore, combining other methods to enhance the efficacy of immune checkpoint therapy is necessary (26), such as small molecule multi-target tyrosine kinase inhibitors and Group A streptococcus preparations. Soft tissue sarcomas are not the optimal tumor type for immune checkpoint therapy, and the development of ICIs is not mature in China. Thus, how to rationally apply ICIs and further integrate them into the comprehensive treatment paradigm for soft tissue sarcomas is a direction that should be pursued in the future, with the hope of bringing more treatment opportunities for patients with advanced soft tissue sarcomas. However, the patient reported poor economic conditions and refused comprehensive immunotherapy.

## Conclusion

A rare case of IS in the left atrium is presented. Cardiac IS, an uncommon malignant tumor of the heart, has an unclear origin, potentially involving a unique pathogenic mechanism. Given its atypical clinical presentation and pathological features, it necessitates heightened attention from clinicians and pathologists to avoid misdiagnosis and mistreatment. Future research should focus on enhancing understanding of this disease, thoroughly investigating its immunopathological and molecular pathological changes, and identifying additional genetic targets and immune checkpoints. These advancements would provide new evidence for the classification, diagnosis, and treatment of cardiac malignancies.

## Data Availability

The original contributions presented in the study are included in the article/Supplementary Material, further inquiries can be directed to the corresponding author.

## References

[1] Crispi F Martinez JM. 94-cardiac tumors. Obstetr Imaging Fetal Diagn Care. (2018):416–8.e1. 10.1016/B978-0-323-44548-1.00094-2

[2] Saraiva J Antunes PE Carvalho L Antunes MJ. Primary malignant cardiac tumors: surgical results. Rev Port Cardiol. (2016) 35(4):199–204. 10.1016/j.repc.2015.11.005 26992743

[3] Basso C Rizzo S Valente M Thiene G. Cardiac masses and tumours. Heart. (2016) 102(15):1230–45. 10.1136/heartjnl-2014-306364 27277840

[4] Travis WD Brambilla E Nicholson AG Yatabe Y Austin JHM Beasley MB WHO Classification of Tumours of the Lung, Pleura, thymus and Heart. 4th ed. Lyon: IARC Press (2015). p. 300–48.

[5] Dewaele B Floris G Finalet-Ferreiro J Fletcher CD Coindre JM Gillou L Coactivated platelet-derived growth factor receptor α and epidermal growth factor receptor are potential therapeutic targets in intimal sarcoma. Cancer Res. (2010) 70:7304–14. 10.1158/0008-5472.CAN-10-1543 20685895

[6] Bode-Lesniewska B Debiec-Rychter M Tavora F. WHO Classification of Tumours Editorial Board. In: Fletcher CDM Bridge JA Hogendoorn PCW Mertens F, editors. WHO Classification of Tumours of Soft Tissue and Bone. Lyon, France: IARC Press (2020). Intimal sarcoma; p. 315–7.

[7] Vinod P Jabri A Hegde V Lahorra J Cutler D. Functional mitral stenosis: imposture of primary cardiac intimal sarcoma. Cardiol Res. (2018) 9(5):307–13. 10.14740/cr748w 30344829 PMC6188046

[8] Burke A. Primary malignant cardiac tumors. Sem Diagn Pathol. (2008) 25(1):39–46. 10.1053/j.semdp.2007.10.006 18350921

[9] Spartalis M Tzatzaki E Spartalis E Moris D Athanasiou A Kyrzopoulos S Primary cardiac intimal sarcoma masquerading as mitral stenosis. Clin Caserep. (2017) 5(8):1422–3. 10.1002/ccr3.1089 PMC5538049 28781875

[10] Chen Y Li Y Zhang N Shang J Li X Liu J Clinical and imaging features of primary cardiac angiosarcoma. Diagnostics (Basel). (2020) 10(10):776. 10.3390/diagnostics10100776 33008011 PMC7600236

[11] Ye N Lan L Hu H Liu J Xu H. Case report: the diagnostic challenge of primary cardiac intimal sarcoma. Front Cardiovasc Med. (2023) 10:1089636. 10.3389/fcvm.2023.1089636 36844745 PMC9947778

[12] Neuville A Collin F Bruneval P Parrens M Thivolet F Gomez-Brouchet A Intimal sarcoma in the most frequent primary cardiac sarcoma: clinicopathologic and molecular retrospective analysis of 100 primary cardiac sarcomas. Am J Surg Pathol. (2014) 38:461–9. 10.1097/PAS.0000000000000184 24625414

[13] Nassereddine H Sciot R Debiec-Rychter M Aydin S Libbrecht L. Cardiac intimal sarcoma: a case report of a rare tumor with peculiar histopathological findings. Ann Pathol. (2019) 39(6):440–3. 10.1016/j.annpat.2019.08.001 31488339

[14] Sciot R. MDM2 Amplified sarcomas: a literature review. Diagnostics. (2021) 11:496. 10.3390/diagnostics11030496 33799733 PMC8001728

[15] Agaram NP Zhang L Sung Y-S Singer S Stevens T Prieto-Granada CN GLI1-amplifications Expand the spectrum of soft tissue neoplasms defined by GLI1 gene fusions. Mod Pathol. (2019) 32:1617–26. 10.1038/s41379-019-0293-x 31189998 PMC6821565

[16] Feeley KP Adams CM Mitra R Eischen CM. MDM2 Is required for the survival and growth of p53-deficient cancer cells. Cancer Res. (2017) 77(14):3823–33. 10.1158/0008-5472.CAN-17-0809 28576884 PMC5523659

[17] Ito Y Maeda D Yoshida M Yoshida A Kudo-Asabe Y Nanjyo H Cardiac intimal sarcoma with PDGFRbeta mutation and co-amplification of PDGFRalpha and MDM2: an autopsy case analyzed by whole-exome sequencing. Virchows Arch. (2017) 471:423–8. 10.1007/s00428-017-2135-x 28474091

[18] Roszik J Khan A Conley AP Livingston JA Groisberg R Ravi V Unique aberrations in intimal sarcoma identified by next-generation sequencing as potential therapy targets. Cancers (Basel). (2019) 11:1283. 10.3390/cancers11091283 31480474 PMC6770224

[19] Giner F Machado I Rubio-Martínez LA López-Guerrero JA Claramunt-Alonso R Navarro S Intimal sarcoma with MDM2/CDK4 amplification and p16 overexpression: a review of histological features in primary tumor and Xenograft, with immunophenotype and molecular profiling. Int J Mol Sci. (2023) 24(8):7535. 10.3390/ijms24087535 37108696 PMC10141691

[20] Aboud A Farha K Hsieh WC Brasch F Ensminger S Gummert J Prognostic factors for long-term survival after surgical resection of primary cardiac sarcoma. Thorac Cardiovasc Surg. (2019) 67(8):665–71. 10.1055/s-0039-1692409 31250414

[21] Coelho PN Banazol NG Soares RJ Fragata JI. Long-term survival with heart transplantation for fibrosarcoma of the heart. Ann Thorac Surg. (2010) 90(2):635–6. 10.1016/j.athoracsur.2010.01.078 20667366

[22] Karavasilis V Seddon BM Ashley S Al-Muderis O Fisher C Judson I. Significant clinical benefit of first-line palliative chemotherapy in advanced soft-tissue sarcoma: retrospective analysis and identification of prognostic factors in 488 patients. Cancer. (2008) 112(7):1585–91. 10.1002/cncr.23332 18278813

[23] Qin S Xu L Yi M Yu S Wu K Luo S. Novel immune checkpoint targets: moving beyond PD-1 and CTLA-4. Mol Cancer. (2019) 18(1):155. 10.1186/s12943-019-1091-2 31690319 PMC6833286

[24] Tawbi HA Burgess M Bolejack V Van Tine BA Schuetze SM Hu J Pembrolizumab in advanced soft-tissue sarcoma and bone sarcoma (SARC028): a multicentre, two-cohort, single-arm, open-label, phase 2 trial. Lancet Oncol. (2017) 18(11):1493–501. 10.1016/S1470-2045(17)30624-1 28988646 PMC7939029

[25] Seligson ND Chen JL Goodrich AC Van Tine BA Campbell JD Richards AL A multicenter, randomized, non-comparative, phase II study of nivolumab ± ipilimumab for patients with metastatic sarcoma (Alliance A091401): expansion cohorts and correlative analyses. J Immunother Cancer. (2024) 12(9):e009472. 10.1136/jitc-2024-009472 39343511 PMC11440204

[26] Pollack SM Redman MW Baker KK Wagner MJ Schroeder BA Loggers ET Assessment of doxorubicin and pembrolizumab in patients with advanced anthra-cycline-naive sarcoma: a phase 1/2 nonrandomized clinical trial. JAMA Oncol. (2020) 6(11):1778–82. 10.1001/jamaoncol.2020.3689 32910151 PMC7489365

